# Hypertensive Rats Treated Chronically With N^ω^-Nitro-L-Arginine Methyl Ester (L-NAME) Induced Disorder of Hepatic Fatty Acid Metabolism and Intestinal Pathophysiology

**DOI:** 10.3389/fphar.2019.01677

**Published:** 2020-01-31

**Authors:** Bo Li, Xinglishang He, Shan-Shan Lei, Fu-Chen Zhou, Ning-Yu Zhang, Ye-Hui Chen, Yu-Zhi Wang, Jie Su, Jing-Jing Yu, Lin-Zi Li, Xiang Zheng, Rong Luo, Dorota Kołodyńska, Shan Xiong, Gui-Yuan Lv, Su-Hong Chen

**Affiliations:** ^1^ Collaborative Innovation Center of Yangtze River Delta Region Green Pharmaceuticals, Zhejiang University of Technology, Hangzhou, China; ^2^ College of Pharmaceutical Science, Zhejiang Chinese Medical University, Hangzhou, China; ^3^ Department of Inorganic Chemistry, Faculty of Chemistry, Institute of Chemical Sciences, Maria Curie-Skłodowska University, Lublin, Poland; ^4^ Institute of Materia Medica, Shandong First Medical University & Shandong Academy of Medical Sciences, Jinan, China

**Keywords:** hypertension, liver injury, multidimensional mass spectrometry-based shotgun lipidomics (MDMS-SL), fatty acid, intestinal pathophysiology

## Abstract

N^ω^-nitro-L-arginine methyl ester (L-NAME), an inhibitor of nitric oxide (NO) biosynthesis, results in hypertension and liver injury. This study aimed at investigating the changes of liver lipometabonomics and exploring the underlying mechanisms of liver injury in the L-NAME-treated rats. The male Sprague-Dawley (SD) rats were treated with L-NAME (40 mg/kg, p.o.) for 8 weeks. After that, the liver, aorta, fecal, and serum were collected for analysis. The results showed that L-NAME induced hypertension and disordered the endothelial nitric oxide synthase (eNOS)-NO pathway in the treated rats. L-NAME could also increase the levels of serum total cholesterol (TC), triglyceride (TG), alanine transaminase (ALT), and aspartate transaminase (AST). The multidimensional mass spectrometry-based shotgun lipidomics (MDMS-SL) analysis showed that L-NAME could induce significant changes of the total hepatic lipids and most hepatic triglycerides, as well as fatty acid (FA). A positive correlation was found between the blood pressure and TAG. Immunofluorescence and Western-Blot experiments indicated that the L-NAME treatment significantly influenced some FA β-oxidation, desaturation, and synthesis-related proteins. The increase of intestinal inflammation, decrease of microcirculation and tight junction proteins, as well as alterations of microbial communities were observed in the L-NAME induced hypertensive rats, as well as alterations of microbial communities were notable correlation to TAG and FA species. This study demonstrated that the L-NAME-induced hypertensive rats exhibiting liver injury were the joint action of hepatic abnormal fatty acid metabolism and microcirculation disorder. Furthermore, the gut microflora, as well as the changes of FA β-oxidation (ACOX, CPT1α), desaturation (SCD-1), and synthesis (FAS) may be the potential mechanisms for abnormal fatty acid metabolism.

## Introduction

Hypertension is one of the most common risk factors affecting cardiovascular disease. Hypertension, or high blood pressure (BP), is defined as a sustained systolic pressure ≥ 130 mmHg or diastolic pressure ≥ 80 mmHg according to the 2017 American College of Cardiology/American Heart Association (ACC/AHA) High Blood Pressure Guideline (Wang et al., 2018). The epidemiologic research shows that the occurrence of hypertension displays a consistent increasing trend. The incidence rate is almost 29.8% in the world (Mills et al., 2015). In China, the prevalence of hypertension, based on the 2017 ACC/AHA guidelines, may be twice incidence at 46.4% (Wang et al., 2018). At least 760 million people worldwide die per year from cardiovascular diseases associated with hypertension, representing 13.5% of the total causes of death (Lawes et al., 2008; Li et al., 2018a).

Multiple research projects have proved that hypertension is associated with severe symptoms of target organ damage (TOD) (Li et al., 2017a), including, but not limited to, complications of the heart, brain, kidney, and eye. The liver is one of the essential metabolic organs in human and also a target organ of multiple metabolic diseases. There is an increasing evidence that hypertension can induce liver injury and lipid metabolic disorder (Lyu et al., 2016; Zhu et al., 2016). The clinical studies have also found out that the prevalence of fatty liver disease (FLD) in patients with hypertension is significantly higher in patients with normal blood pressure and is approximately twice as large as patients without FLD (Yehua and Xiaowa, 2000). Our previous study results showed that the levels of serum total cholesterol (TC), triglyceride (TG), alanine transaminase (ALT), and aspartate transaminase (AST) in the spontaneously hypertensive rats (SHR) were significantly increased (Chen et al., 2015). These results suggest that hypertension is closely related to the occurrence of liver injury. Therefore, it is vital to study the association of hypertension induction and related liver injury.

Essential, or primary hypertension is caused first of all by endothelial dysfunction, which results from nitric oxide (NO) deficiency (Bilanda et al., 2017). The functions of NO, both injurious and protective, are involved in the development of liver injury and L-Arginine, a precursor of NO, can attenuate liver injury (Clemens, 1999; Angele et al., 2000). N^ω^-nitro-L-arginine methyl ester (L-NAME) is an inhibitor of NO biosynthesis blocking the activity of nitric oxide synthase (NOS) and inducing hypertension (Tarsitano et al., 2007; Rajeshwari et al., 2014; Seth et al., 2016; Chen et al., 2019). It has been found out that L-NAME-induced NO deficiency in hypertensive rats can cause liver injury and vacuolation (Rajeshwari et al., 2014), as well as changes in the serum lipid profiles (Abd-Elsalam et al., 2017; Bilanda et al., 2017), but lack of research on liver lipids species changes that are closely related to liver injury.

Lipidomics can detect minor changes in various lipid molecules (Huang et al., 2019). Mass spectrometry, chromatography-mass spectrometry, and other techniques are widely used in lipid research (Gupta et al., 2018). Multidimensional mass spectrometry-based shotgun lipidomics (MDMS-SL), based on the multi-dimensional mass spectrometry, is currently recognized as one of the most rapid and effective methods for lipid analysis and is used to study many diseases (Yang et al., 2009; Hu et al., 2015; Hu et al., 2016; Huang et al., 2019). This method can make the lipid extract directly from the injection system into an electrospray ionization mass spectrometry (ESI-MS) without chromatographic separation (Yang et al., 2009; Hu et al., 2015; Hu et al., 2016; Huang et al., 2019). In the metabolic pathway, FA in liver involves fatty acid uptake, desaturase, oxidative decomposition, and synthesis, including related metabolic enzymes or proteins of fatty acid transporter-α (FATP2) (Krammer et al., 2011), stearoyl-CoA desaturase-1 (SCD-1), acyl-CoA oxidase 1 (ACOX1) (Shinohara et al., 2012; Huang et al., 2019), carnitine palmitoyltransferase-1α (CPT1α) (Huang et al., 2019), and fatty acid synthase (FAS) in the liver, respectively. In this study MDMS-SL was used to investigate the changes in liver lipids and further investigations were carried out to determine whether the metabolic enzymes were involved in the underlying mechanisms of fatty acid metabolism in the L-NAME-treated rats.

There is increasing evidence of the role of host-gut interactions on hypertension and liver injury (Santisteban et al., 2017; Albillos et al., 2019). Multiple studies have demonstrated a link between the gut dysbiosis and hypertension in rats, mice, and humans (Yang et al., 2015; Li et al., 2017b; Santisteban et al., 2017). These changes in gut pathology, with the increasing intestinal permeability, reduced tight junction proteins, decreased gut perfusion, and gut microbial dysbiosis, were associated with alterations in the microbial communities involved in blood pressure control in the SHR and chronic angiotensin II infusion rat models (Santisteban et al., 2017). Also, more and more studies have shown that the changes of intestinal bacteria can affect liver lipid metabolism, and that probiotics intervention reduce liver disease (Albillos et al., 2019). Therefore, we speculated that L-NAME-induced gut microflora disorder in hypertensive rats may lead to liver lipid metabolism disorder, further aggravating liver damage.

In this study, the Sprague-Dawley (SD) rats chronically treated with L-NAME were used to prepare a hypertensive rat model and to observe the microcirculation, pathology, as well as ultrastructural changes of the liver and gut tissues. MDMS-SL was applied to analyze the changes of hepatic lipid metabolism, mainly triglycerides (TAG), fatty acids (FAs), and phosphatidylcholine (PC) in the L-NAME-treated rats. Additionally, it was investigated whether the metabolic enzymes involved in the underlying mechanisms of fatty acid metabolism in the liver were altered in response to the L-NAME interventions. Moreover, this study was designed to test the dysbiosis in the gut microbiota associated with hypertension related liver injury caused by reduced NO content.

## Materials and Methods

### Chemicals and Reagents

Total cholesterol (TC), triglyceride (TG), alanine transaminase (ALT), and aspartate transaminase (AST) kits were all purchased from Medical System Biotechnology Co., Ltd. (Ningbo, Zhejiang, China). Hematoxylin-Eosin solution, 3,3’-Diaminobenzidine (DAB) solution, and nitric oxide (NO) assay kit were all purchased from Nanjing Jiancheng Technology Co., Ltd. (Jiangsu, China). Oil red O solution was form BBI Life Sciences Corporation (Zhejiang, China). Antibodies against carnitine palmitoyltransferase-1α (CPT1α), fatty acid transporter-2 (FATP2), stearoyl-CoA desaturase-1 (SCD-1), acyl-CoA oxidase 1 (ACOX1), fatty acid synthase (FAS), occludin, HRP conjugated goat anti-rabbit IgG (H+L), FITC conjugated goat anti-rabbit IgG (H+L), and β-actin were purchased from Proteintech Group (Proteintech, USA). HRP conjugated goat anti-mouse/rabbit IgG (PV-6001) was from Zhongshan Goldenbridge Biotechnology Co., Ltd. (Beijing, China). The BCA protein concentration determination kit, RIPA lysate kit, DAPI staining solution, and the chemiluminescent assay kit were from Beyotime Biotechnology (Jiangsu, China).

N^ω^-nitro-L-Arginine Methyl Ester (L-NAME) was from Yuanye Biotechnology Co., Ltd. (Shanghai, China). All triheptadecenoyl glycerol (T17:1 TAG) was purchased from Nu Chek, Inc. (Elysian, MN, USA). All other reagents were at least analytical grade and purchased from Fisher Scientific (Pittsburgh, PA), Sigma-Aldrich Chemical Company (St. Louis, MO), or as specified.

### Animals and Experimental Design

Sprague-Dawley Rats (Male) were obtained from Zhejiang Academy of Medical Science (SCXK 2014-0001, Hangzhou, China). The animal operations were in accordance with the Guidelines of Care and Use of Laboratory Animals of Zhejiang University of Technology and the operations were approved by the Ethics Committee of Zhejiang University of Technology.

Eighteen SD rats were divided into the normal group (NG) and the L-NAME-treated group (L-NAME), with nine rats in each group. During the experiment, all rats were fed with the standard diet. The normal group rats were given purified water every day, and the L-NAME group was given L-NAME (40 mg/kg, p.o.) with the volume 1 ml/100 g according to the weight for 8 weeks. At the end of the experiment, the rats were anesthetized and the liver, aorta, and gut tissues were separated as soon as possible. The tissues parts were put into the 10% formalin, 2.5% glutaraldehyde, and 30% sucrose solutions for hepatic pathology. The other parts were stored at −80˚C for the hepatic lipids and Western-Blot analysis.

### Non-Invasive Measurement of Blood Pressure

The blood pressure (BP) was monitored within 1, 3, and 7 weeks. The BP-2010 AUL non-invasive blood pressure monitoring system (Softron Biotechnology, Beijing, China) was used to measure BP of SD rats as described previously (Chen et al., 2015). Briefly, the air tightness of the instrument monitoring channel was examined, the temperature of the incubator was adjusted to 37°C, and kept at room temperature. The rats were then placed in a monitoring environment for 15 min in the fixed bag. Meanwhile, the BP monitoring sensors were round the tail root of rats. When the signal was stable, the data of systolic blood pressure (SBP), diastolic blood pressure (DBP), and mean arterial blood pressure (MBP) were detected and collected.

### Determination of Serum Transaminase and Lipid Levels

After treatment for 4 and 6 weeks, the rats were fasted overnight and the blood was taken from the ophthalmic venous plexus, centrifuged at 3,500 rpm/min for 10 min to collect serum. The serum transaminase (ALT and AST) and the serum lipid (TC and TG) levels were measured with the corresponding kits by an automatic biochemical analyzer (HITACHI-7020, Japan).

### Determination of Serum NO Level

The serum nitric oxide (NO) level was evaluated by the nitrate reductase method as described earlier (Chen et al., 2015). The blood sample was centrifuged at 3,000 rpm/min for 10 min and the serum was separated to determine the NO concentration at the end of experiment. The reductase nitrate restores all NO^3-^ to NO^2-^, which can be determined by the excellent colorimetric reagent to measure the total nitrite as an indicator of NO production. All the procedures were performed as described in the assay kit.

### Hepatic and Intestinal Gross Microcirculation and Microvasculature Observation

For gross microcirculation of the liver and intestine, the microcirculatory blood flow was assessed by laser Doppler with the moor FLPI V2.1 software (Moor Instruments Ltd, Millwey, Axminster, Devon, UK). Firstly, the rats were anaesthetized with 10% chloral hydrate, then the abdominal cavity was exposed and the liver was separated on the transparent plastic sheet. Next the distance of the scanner and liver was set to be 13 cm. One 1.4 cm × 1.4 cm area was selected on the liver. Then the distance of the scanner and liver was set to be 13 cm. The reflective signal of laser beam was received by a detector positioned in the scanner head and converted into the electrical signal to represent the blood perfusion condition (Li et al., 2018a). Detection of gross microcirculation in the intestine is the same as in the liver.

After the observation of gross microcirculation, microcirculatory blood flow and microcirculation of the intestine and liver were assessed by microcirculation in the vivo of tracking and observing systems (Gene&I, Beijing, China). Immediately after putting the cotton sheet round the intestine on the transparent plastic sheet, it was placed on the platform of the inverted biomicroscopy (Nikon) with a constant 37°C, and kept wet. Microvessels were selected with a 10-fold eyepiece. Then they were replaced with the eyepiece with 20-fold. The microcirculation was observed and recorded with the CCD color camera connected with the microscope for 1 min, then the eyepiece was 40-fold converted, and the high-speed camera (IDT) connected with the microscope was used to record for 30 s at a speed of 500 frames/s. Then the microcirculatory blood flow and microcirculation of the liver was observed as the intestine.

### Hematoxylin-Eosin (H&E) Staining and Oil Red O Staining for the Histopathology

An appropriate amount and parts of liver, aorta, intestine, and colon tissues were fixed in 10% formalin. The tissue wax was prepared by the gradient ethanol dehydration, xylene transparency, and paraffin embedding. Tissue sections were prepared for 4 μm and stained with the hematoxylin-eosin (H&E) staining according to the previous literature reports (Lei et al., 2019; Li et al., 2019).

The liver tissue was dehydrated with the 30% sucrose solution before embedding into the OCT compound (Sakura, Tokyo, Japan), and cut into 10 μm frozen sections. Oil red O (OR) staining was made according to the procedure described in our papers (Lei et al., 2019; Li et al., 2019). Briefly, the sections were rinsed and then kept in 60% isopropanol, finally stained with the 0.5% Oil red O solution for 15 min, and in 60% isopropanol for differentiation. Then the nuclei of cells were processed for hematoxylin staining. All H&E and OR staining was photographed with the biological microscope (BX43, Olympus, Japan) at a total magnification of ×400 and analyzed with the Image-Pro Plus software.

### Transmission Electron Microscopy (TEM)

The liver and intestine ultrastructures were observed using the transmission electron microscopy (TEM) as described previously (Lei et al., 2019). Briefly, the livers and intestines were cut into 1 mm^3^ spaced pieces, fixed in 2.5% glutaraldehyde phosphate buffer (pH 7.4), and postfixed in 1% osmium tetroxide. Then the tissues were dehydrated in a graded ethanol series, followed by embedding in the epoxy resin for blocks preparation. The ultrathin sections were cut into 50~70 nm with the ultrathin microtome. The liver ultrastructures were observed using the transmission electron microscopy (HT7700, HITACHI) at a total magnification of ×1200.

### Immunohistochemistry (IHC) Analysis of eNOS in the Aorta and Occludin in the Intestine and Colon

The immunohistochemistry (IHC) staining was similar to that described previously (Li et al., 2018a). The expression and localization of endothelial nitric oxide synthase (eNOS) in the aorta and occludin in the intestine and colon were determined. The deparaffinized tissue sections were incubated with the corresponding primary antibody eNOS or occludin (1:200, dilution). A secondary antibody (HRP conjugated goat anti-mouse/rabbit IgG) was added. The signals were visualized by DAB staining and the nuclei were counterstained with hematoxylin. Positive staining revealed the yellow color under the microscope.

### Immunofluorescence Observation

For the immunofluorescent labeling, the deparaffinized tissue sections were incubated with antibodies against CPT1α, FATP2, SCD-1, ACOX1, and FAS overnight at 4°C after antigen retrieval and blocked. The secondary antibody FITC conjugate goat anti-rabbit IgG (H+L) was added and incubated in a light-tight shield. Then the nuclei were incubated with DAPI for 5 min at room temperature in a light-tight shield. The experiment was performed following the manufacturer’s protocol and were photographed with the laser scanning microscope (Olympus, Japan) at a total magnification of ×400. The data of protein expressions were semi-quantitatively analyzed as the integrated option density (IOD) in the positive area of the microphotograph with the Image-Pro Plus software.

### Preparation of Lipid Extracts From the Liver Samples and MS Analysis of Lipids

A powder sample (~25 mg) from each liver tissue was weighed and further homogenized in 0.5 ml of ice-cold, diluted PBS using sonification (Branson Ultrasonic Bath). A protein assay was performed on individual homogenates applying a bicinchoninic acid protein assay kit (Pierce, Rockford, IL) using the bovine serum albumin as the standard. Lipids were extracted from each sample by employing a modified procedure of Bligh and Dyer extraction in the presence of internal standards that were in a premixed solution for the global lipid analysis and added into each liver tissue sample based on its protein content. Therefore, the lipid levels of liver samples can be normalized to the protein concentration and directly quantified. The lipid extracts were dried with nitrogen, redissolved in 1:1 CHCl_3_/MeOH with a volume of 200 μl/mg protein (present in the original sample) capped, and stored at −20°C for the mass spectrometric (MS) analysis as previously described (Yang et al., 2009).

The MS analyses of lipids and data processing were based on the literature (Hu et al., 2015; Hu et al., 2016). In brief, a triple-quadrupole mass spectrometer (Thermo TSQ Quantiva) equipped with an automated nanospray ion source (TriVersa NanoMate, Advion Bioscience Ltd.) and operated using the X calibur system software, was used in the study. Identification and quantification of different lipid classes and individual species were conducted by MDMS-SL as described based on the lipidomics principles, mainly including triglycerides (TAG), fatty acids (FAs), and phosphatidylcholine (PC).

### Western-Blot Analysis of Fatty Acid Metabolism Nodes

In brief, appropriate liver tissues were lysed at 4°C in the RIPA buffer. The tissue homogenates were centrifuged at 12,000 rpm/min for 15 min at 4°C, and the resulting supernatants were used for the Western-Blot analysis. The total proteins in the supernatants were determined by the BCA assay. Then SDS-PAGE was used to transfer the protein onto the nitric acid fiber membrane. The protein was sealed with the 5% skimmed milk powder, and the first antibody (5% milk, β-actin 1:10,000, CPT1α 1:2,000, FATP2 1:1,000, FAS 1:1,000, and ACOX1 1:1,000) incubated the membrane at 4°C overnight. After being washed three times with TBST, the membrane was incubated with the second antibody, HRP conjugated goat anti-rabbit IgG (H+L) (1:5,000 dilution), for 120 minutes. Finally, the blotted protein bands were detected by the chemiluminescent assay kit and the protein expression levels were normalized to β-actin by densitometry with the Image J software.

### 16S rRNA Amplification of V3-V4 Region and Illumina Sequencing

The fecal bacterial analysis was evaluated by 16S rRNA amplification of V3-V4 region and Illumina sequencing as previously described (Lei et al., 2019). Briefly, the V3-V4 hypervariable regions of the bacteria 16S rRNA gene were amplified with primers 338F (5′-ACTCCTACGGGAGGCAGCAG-3′) and 806R (5′-GGACTACHVG GGTWTCTAAT-3′) by the thermocycler PCR system (GeneAmp 9700, ABI, USA). The resulted PCR products were extracted from a 2% agarose gel and further purified using the AxyPrep DNA Gel Extraction Kit (Axygen Biosciences, Union City, CA, USA) and quantified using QuantiFluor™-ST (Promega, USA) according to the manufacturer’s protocol. The purified DNA from the individual samples was sequenced by the Illumina MiSeq platform (Illumina, San Diego, USA) according to the standard protocols by Major bio Bio-Pharm Technology Co. Ltd. (Shanghai, China). The operational taxonomic units (OTUs) were clustered with 97% similarity cutoff using UPARSE (version7.1 http://drive5.com/uparse/) and chimeric sequences were identified and removed using UCHIME. The taxonomy of each 16S rRNA gene sequence was analyzed by the RDP Classifier algorithm (http://rdp.cme.msu.edu/) against the Silva (SSU123) 16S rRNA database using the confidence threshold of 70%. The data were scoped on the I-Sanger cloud platform (http://www.i-sanger.com) developed by Majorbio Bio-Pharm Technology Co. Ltd. The community of generic level as well as the calculated alpha diversity (Chao1) and the beta diversity on the generic level were analyzed. Statistical comparisons of the results were performed using the Student’s t-test.

### Statistical Analysis

All data were expressed as the means ± SEM and subjected to the *t*-test. When compared with the same period of the model group, the data were subjected to the independent-sample *t*-tests. The two-sided Student’s t-test were used to examine differences in microflora between two groups. The Pearson correlation coefficient was used in the examination of correlations between the SBP level and the TAG species. The Spearman correlation coefficient was used to examine the correlations between the relative abundance of the gut microbial community at the genus level and the TAG and FA species, as well as the serum NO and liver microcirculation. All analyses were performed using the updated version of SPSS software. Diagrams were prepared applying the Graph Prims and Excel software.

## Results

### L-NAME Induced Hypertension and eNOS/NO Pathway Imbalance

**Figure 1:**
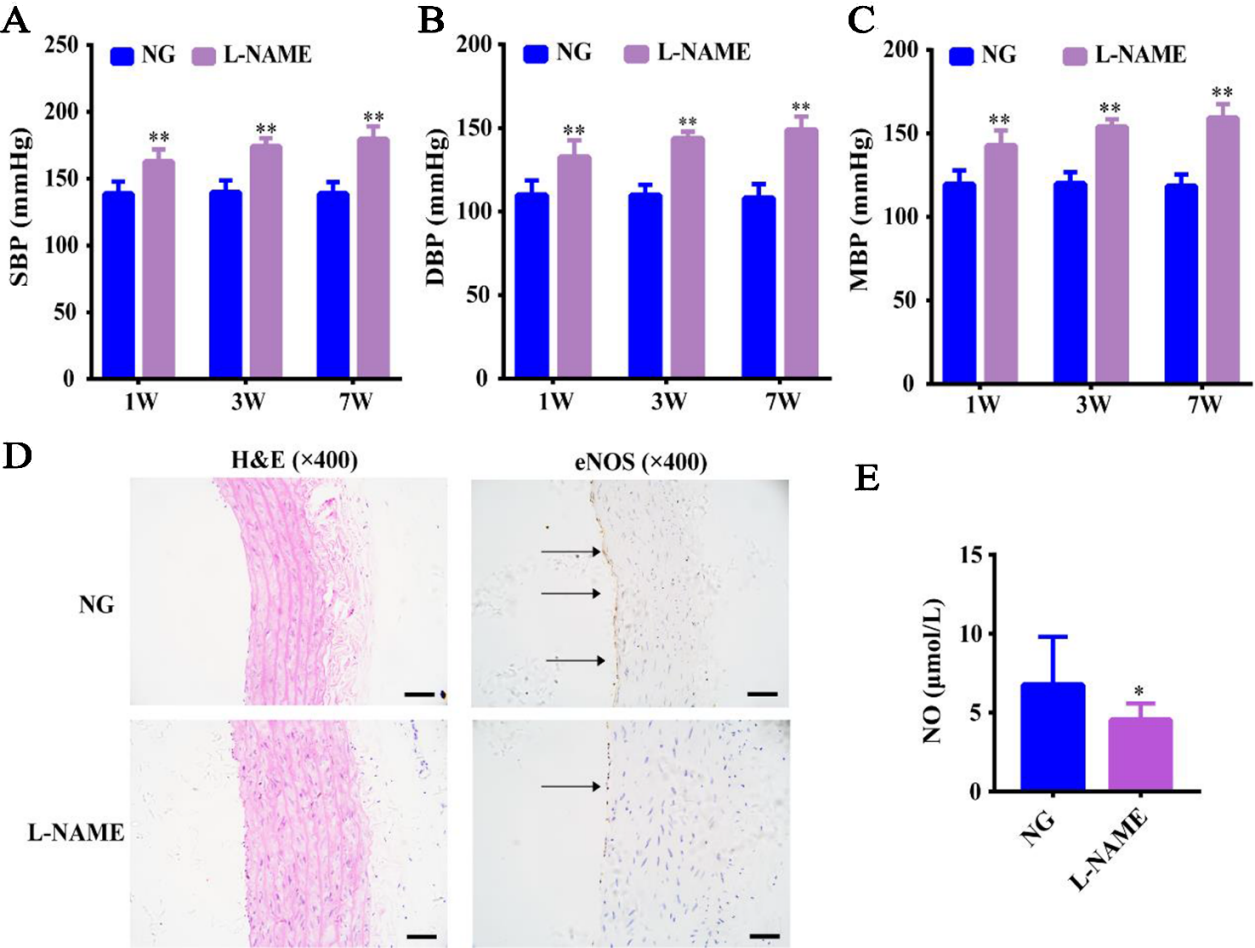
L-NAME induced hypertension and eNOS-NO pathway imbalance without an effect on the weight gain. **(A)** Changes of systolic blood pressure (SBP). **(B)** Changes of diastolic blood pressure (DBP). **(C)** Changes of mean arterial blood pressure (MBP). **(D)** Representative photomicrograph of histological alterations in aorta (×400) and endothelial nitric-oxide synthase (eNOS) protein expression by immunohistochemistry (IHC) in aorta (×400) (the arrows mean the expression of eNOS). **(E)** Nitric oxide (NO) content in serum. Bar = 50 μm. The data were expressed as means ± SEM. Significant differences are indicated by *P < 0.05, **P < 0.01 as compared with the NG (n = 9 per group).

Compared with the NG, there was no significant effect on the body weight of the L-NAME-treated rats continuously molded for 7 weeks (data not shown), which indicated that L-NAME did not affect the normal growth of rats. SBP, DBP, and MBP in the L-NAME-treated rats were increased by 14.8, 17.1, and 16.2%, respectively compared with the normal rats after the 1-week modeling. The increased rates of SBP, DBP, and MBP were 19.8%, 23.6%, 22.1% after the 3^rd^ week and went up by 22.7%, 27.4%, 25.6% after the 7^th^ week (**Figures 1A–C**) (*P <* 0.01). This indicated that increasing the exposure time results in greater increase rate was observed in the blood pressure of rats.

Further, we also found that the expression of eNOS in aorta endothelium was significantly reduced with thickening of vascular wall (**Figure 1D**), and L-NAME caused a significant decrease of serum NO level in rats (**Figure 1E**) (*P* < 0.05). This suggested that the increased BP may be related to the endothelial nitric oxide synthase-nitric oxide (eNOS-NO) pathway imbalance in the L-NAME-treated rats.

### L-NAME Induced Changes of Serum Lipid Profiles and Liver Injury

**Figure 2:**
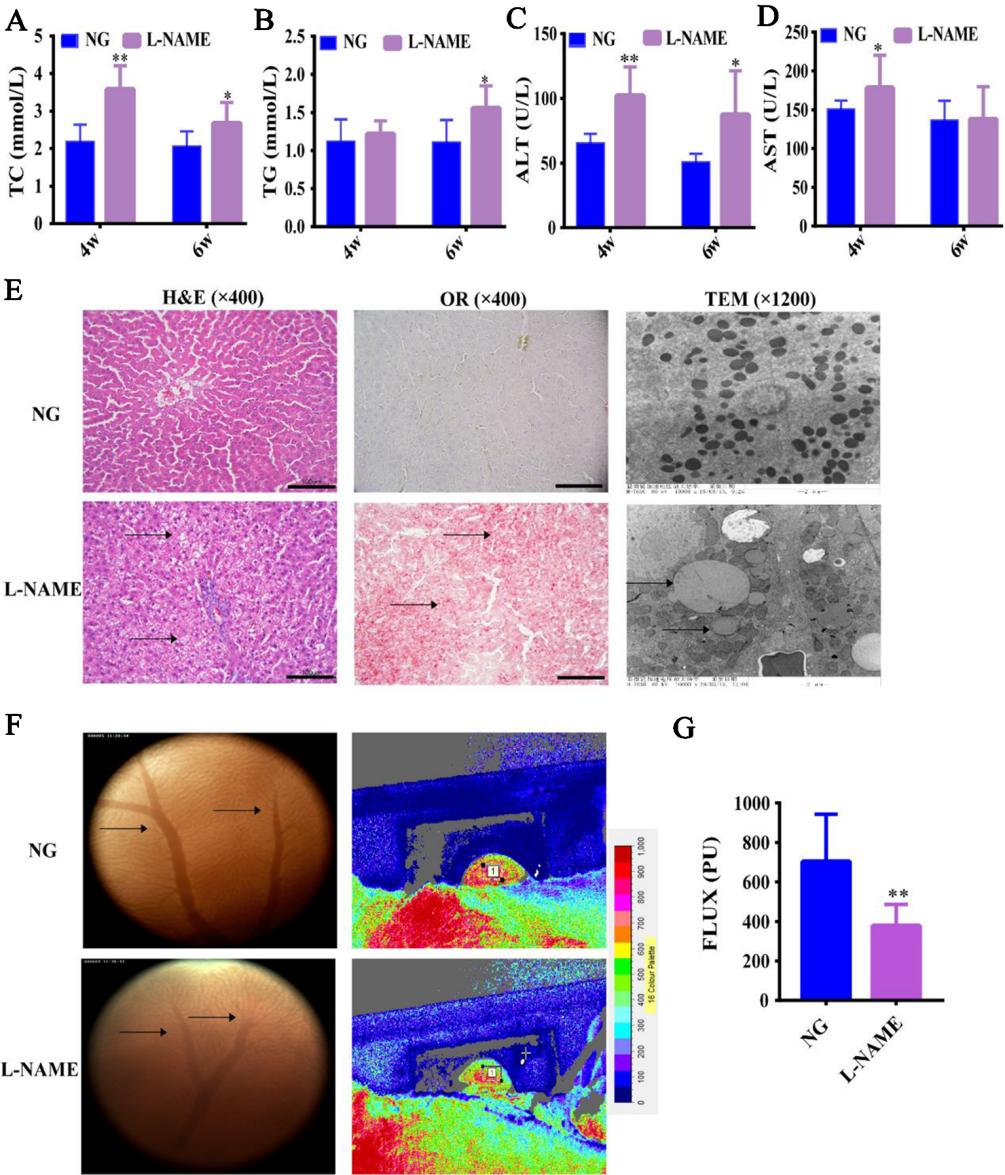
Changes of serum lipid profiles, liver function, and hepatic histopathology in the L-NAME-treated rats. **(A)** Changes of serum TC. **(B)** Changes of serum TG. **(C)** Changes of serum AST. **(D)** Changes of serum ALT. **(E)** Representative photomicrograph of histological alterations in the liver by H&E staining (×400), Oil red O staining (×400), and transmission electron microscopy (×1,200) (the arrows indicate the lipid accumulation in the liver). **(F)** Changes of gross microcirculation and hepatic microcirculation (the arrows indicate the microvessels in the liver). **(G)** The gross microcirculation perfusion. Bar = 100 μm. The data were expressed as means ± SEM. Significant differences are indicated by *P < 0.05, **P < 0.01 as compared with the NG (n = 9 per group).

Firstly, in order to confirm that L-NAME-induced NO deficiency in hypertensive rats can cause liver injury, as well as changes in the serum lipid profiles. In contrast to the NG, the levels of serum TC, ALT, and AST in the L-NAME-treated rats were significantly increased after the 4^th^ week, and the levels of serum TG and ALT after the 6^th^ week (**Figures 2A–D**) (*P <* 0.01, 0.05). Moreover, the ALT level increase rate was 58% after the 4^th^ week and increased by 72% after the 6^th^ week compared with the NG. The results suggested that there might be abnormal lipids metabolism and liver injury in the L-NAME-treated rats, which was consistent with other studies (Abd-Elsalam et al., 2017; Bilanda et al., 2017).

Further study of the effect of L-NAME on the liver, we observed the hepatic gross microcirculation and microvasculature abundance to assess the change of hepatic microcirculatory perfusion. Compared with the NG, L-NAME could induce significant decline of perfusion in the liver with a decrease in the number of microvessels (**Figures 2F, G**) (*P <* 0.01). H&E staining and OR staining revealed that there was a wide range of abnormal lipid accumulation in the liver of the L-NAME-treated rats (**Figure 2E**). Besides, the ultrastructural analysis of hepatocytes with TEM showed that in the normal hepatocytes, the nucleus was round and central, the nuclear membrane was clear, and the rough endoplasmic reticulum was abundant and closely arranged with numerous mitochondria. While the L-NAME-treated rats exhibited significant lipid droplets accumulation, mitochondrial damage, and disordered endoplasmic reticulum structure (**Figure 2E**). These data indicated that there were abnormal hepatic microcirculation and histopathology in the rats chronically treated with L-NAME.

### Changes of Hepatic Lipometabonomics

**Figure 3:**
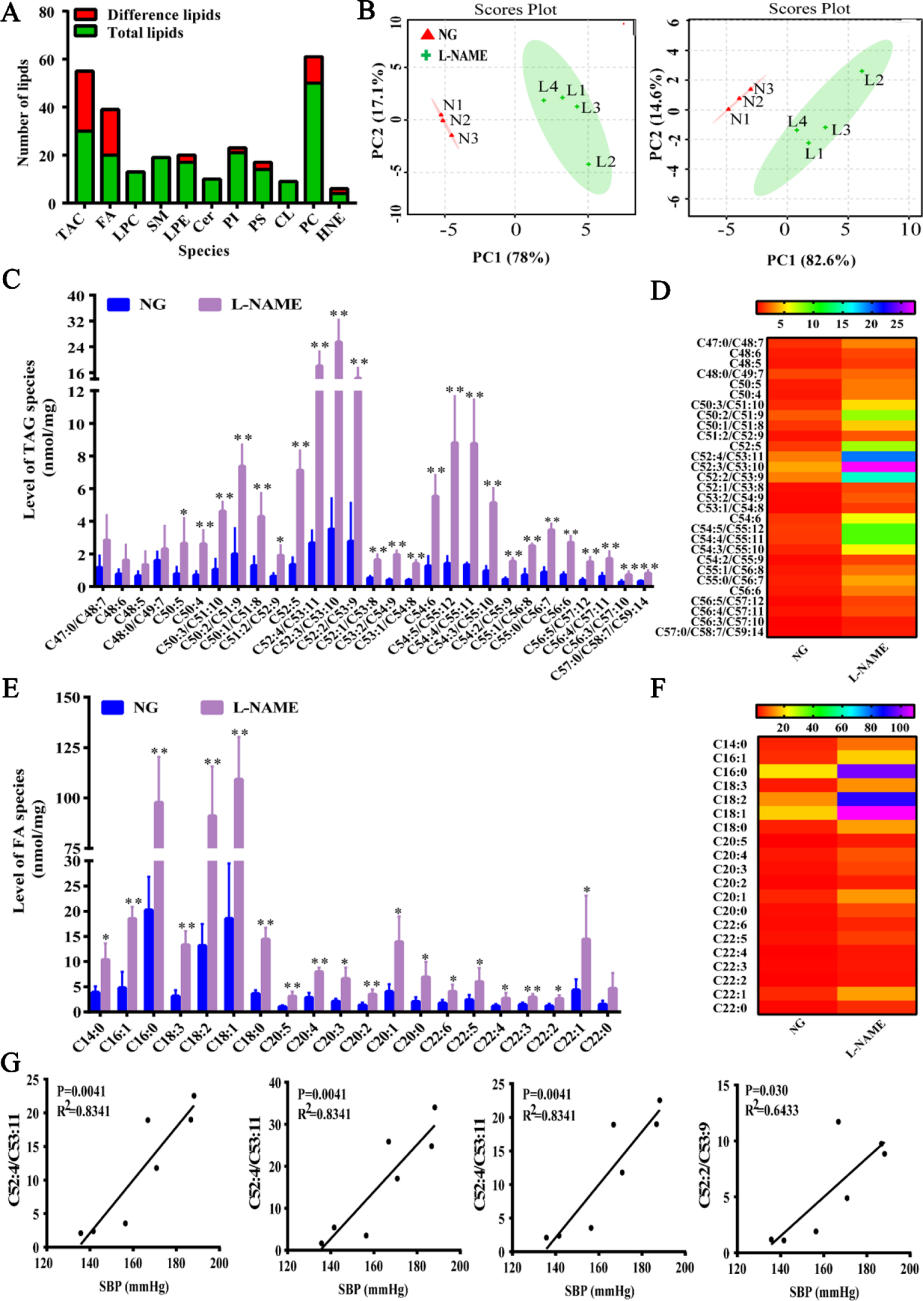
Changes of total liver lipids, triglycerides, and fatty acids of liver in the L-NAME-treated rats. **(A)** Changes of a number of lipids. **(B)** PCA of TAG (left) and FA (right) in the liver. **(C)** Mass level of TAG species in the liver. **(D)** Heat map of TAG in the liver. **(E)** Mass level of FA species in the liver. **(F)** Heat map of FA in the liver. **(G)** Correlation analysis between the top four contents of TAG and SBP in the liver of rats. The data were expressed as means ± SEM. Significant differences are indicated by *P < 0.05, **P < 0.01 as compared with the NG (n = 3~4 per group).

In the present study, it was found that NO deficiency could cause the liver lipid droplets accumulation and liver injury, but there was no evidence whether L-NAME-induced NO deficiency could induce disorder of lipid molecules, which is closely related to liver injury. So, the method of MDMS-SL was used to investigate these changes. The total of 207 lipid molecules were detected by MDMS-SL, including 30 kinds of triglycerides (TAG), 20 kinds of fatty acids (FAs), 10 kinds of ceramide (Cer), 21 kinds of phosphatidylinositol (PI), 50 kinds of phosphatidylcholine (PC), 13 kinds of lysophosphatidylcholine (LPC), 17 kinds of lysophosphatidic (LPE), 19 kinds of sphingolipid (SM), 9 kinds of cardiolipin (CL), as well as smaller amount of other lipid molecules. Among them, the most abundant were PC and TAG. Compared with the NG, 65 kinds of lipids significant changes were found after 8 weeks of L-NAME modeling, which indicated that L-NAME could cause the liver lipid metabolism disorder (**Figure 3A**). The principal component analysis (PCA) describes the largest variation in the data using a few orthogonal latent variables. PCA was carried out and the score plot was obtained for the TAG and FA data, including trends and groupings. For TAG and FA, the PC1 separated the samples with respect to the hepatic lipid levels and accounted for 78% and 82.6% of the total variance, respectively, whereas the PC2 accounted for 17.1% and 14.6%, respectively (**Figure 3B**).

There were 30 types of TAG and 20 FA specifically detected in the liver. The total content of TAG in the L-NAME-treated rats was significantly increased (*P < *0.01), from 31.60 ± 2.82 in the NG rats to 144.74 ± 15.02 nmol/mg protein, increasing of 358%. Except for C47:0/C48:7, C48:6, C48:5, and C48:0/C49:7, the other lipids of TAG were significantly increased (**Figures 3C, D**) (*P <* 0.05, 0.01). Further analyses of the correlation between the blood pressure and the top four contents of TAG indicated that SBP was positively correlated with the main lipid content, showing a significant difference (**Figure 3G**). As FA are an important component of TAG, we further analyzed long-chain fatty acid content to accurately analyze changes in TAG species. The results showed that the total FA content in the model group increased significantly (*P* < 0.01), which increased from 94.81 ± 8.46 in the NG to 434.23 ± 45.07 nmol/mg protein, with an increase of 358%. Except for C22:0, other FA were all significantly increased (**Figures 3E, F**) (*P <* 0.05, 0.01). These data suggested that there was an abnormal hepatic fatty acid metabolism in the rats treated chronically with L-NAME.

**Figure 4:**
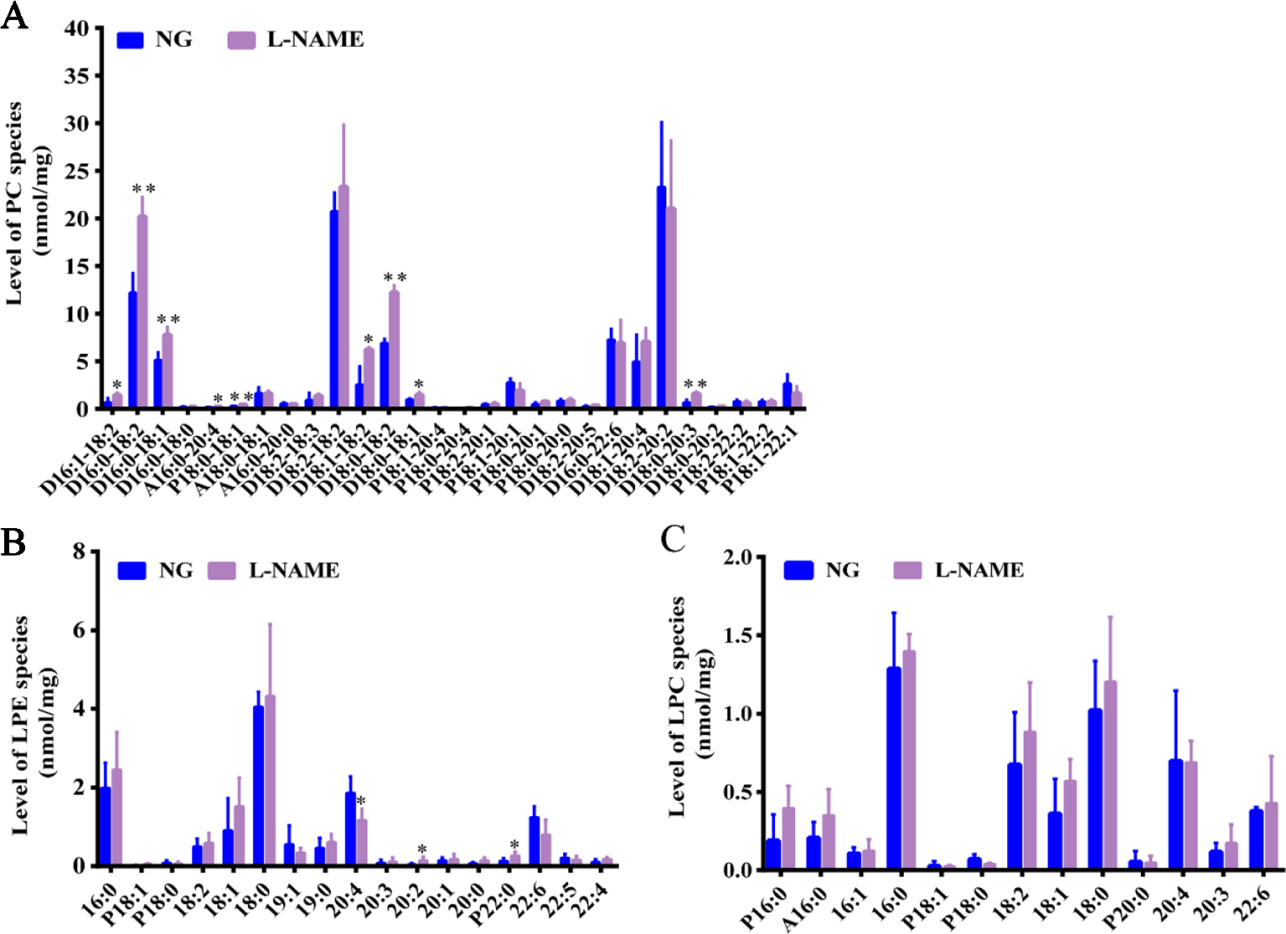
Slight changes of hepatic phospholipid classes in the L-NAME-treated rats. **(A)** Mass level of PC species in the liver. **(B)** Mass level of LPE species in the liver. **(C)** Mass level of LPC species in the liver. The data were expressed as means ± SEM. Significant differences are indicated by *P < 0.05, **P < 0.01 as compared with the NG (n = 3~4 per group).

In the study, for further evaluation of lipids changes in the L-NAME induced rats, the MDMS-SL was also employed to determine the amounts of other phospholipid classes including PC, LPC, and LPE. Compared with the control group, the MDMS-SL analysis showed significant changes of the amount of many PC species, D16:1–18:2, D16:0–18:2, D16:0–18:0, A16:0–20:4, P18:0–18:1, D18:1–18:2, D18:0–18:2, D18:0–18:1, and D18:0–20:3 in the L-NAME-treated rats (**Figure 4A**) (*P <* 0.05, 0.01). At the same time, there were some LPE species, 20:4, 20:2, and P22:2 significant changed (**Figure 4B**) (*P < *0.05). The further analysis demonstrated that the LPC species did not change significantly in the L-NAME-treated rats (**Figure 4C**). These data suggested that L-NAME treatment might slightly alter hepatic levels of phospholipid classes, which also indicated that the L-NAME treatment might induce mainly changes of hepatic fatty acid metabolism.

### Hepatic Fatty Acid Metabolism Related Enzyme Disordered

**Figure 5:**
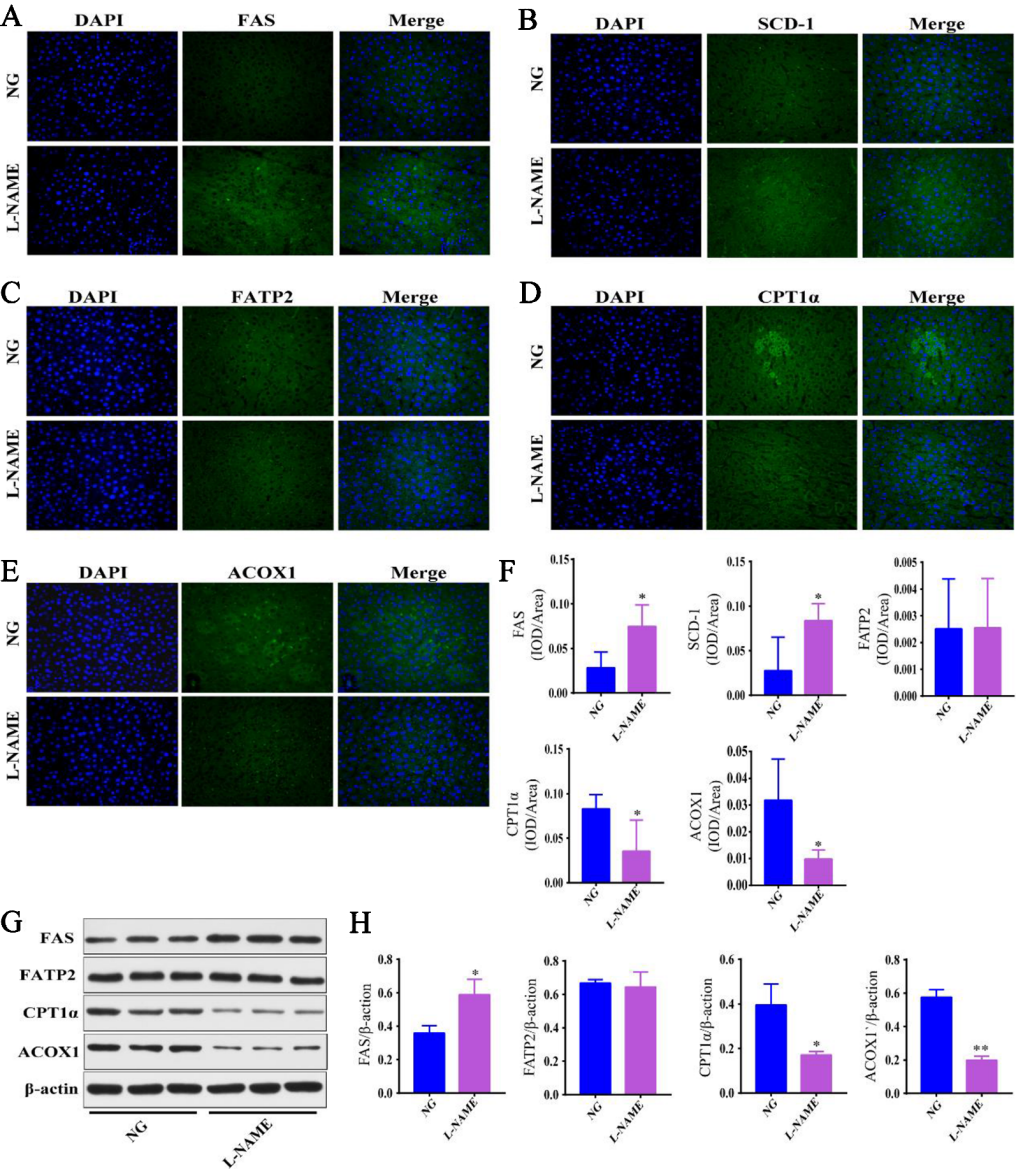
Fatty acid metabolism related enzyme disordered in the L-NAME-treated rats. **(A**–**E)** Representative photomicrograph of FAS, SCD-1, FATP2, CPT1α, and ACOX1 expression in the liver by IHC (×400), respectively. **(F)** Semi-quantitative analysis of the FAS, SCD-1, FATP2, CPT1α, and ACOX1 expression in the liver by IHC. **(G**, **H)** FAS, FATP2, CPT1α, and ACOX1 expression in the liver by the Western-Blot analysis. The data were expressed as means ± SEM. Significant differences are indicated by *P < 0.05, **P < 0.01 as compared with the NG (n = 3 per group).

Further investigations were carried out to determine whether the metabolic enzymes were involved in the underlying mechanisms of hepatic fatty acid metabolism in the L-NAME-treated rats. Some of the proteins involved in the fatty acid synthesis, desaturation, uptake, and oxidation in the liver were measured with the immunofluorescence and Western-Blot method (**Figure 5**). The results showed that the expression of FAS and SCD-1, the key rate limiting enzyme in the synthesis and desaturation of FA, was significantly enhanced in the liver of the L-NAME-treated rats (**Figures 5A, B, F**), the result of FAS corresponded to the Western-Blot assay (**Figures 5G, H**) (*P < *0.05). The FA lipidomics showed that the components of saturated FA and monounsaturated FA increased significantly, and the expression of fatty acid β-oxidation related enzymes ACOX1 and CPTA1α was significantly decreased after the L-NAME treatment (**Figures 5D–F**), the results corresponded to Western-Blot assay (**Figures 5G, H**) (*P <* 0.05, 0.01). Moreover, the expression of FATP2 did not show significant changes. These results indicated that the L-NAME treatment might increase the fatty acid synthesis and desaturation, as well as reduce β-oxidation in the liver, which may be essential factors in regulating the hepatic fatty acid metabolism.

### Gut Pathophysiological Alterations and Microbiota Dysbiosis

**Figure 6:**
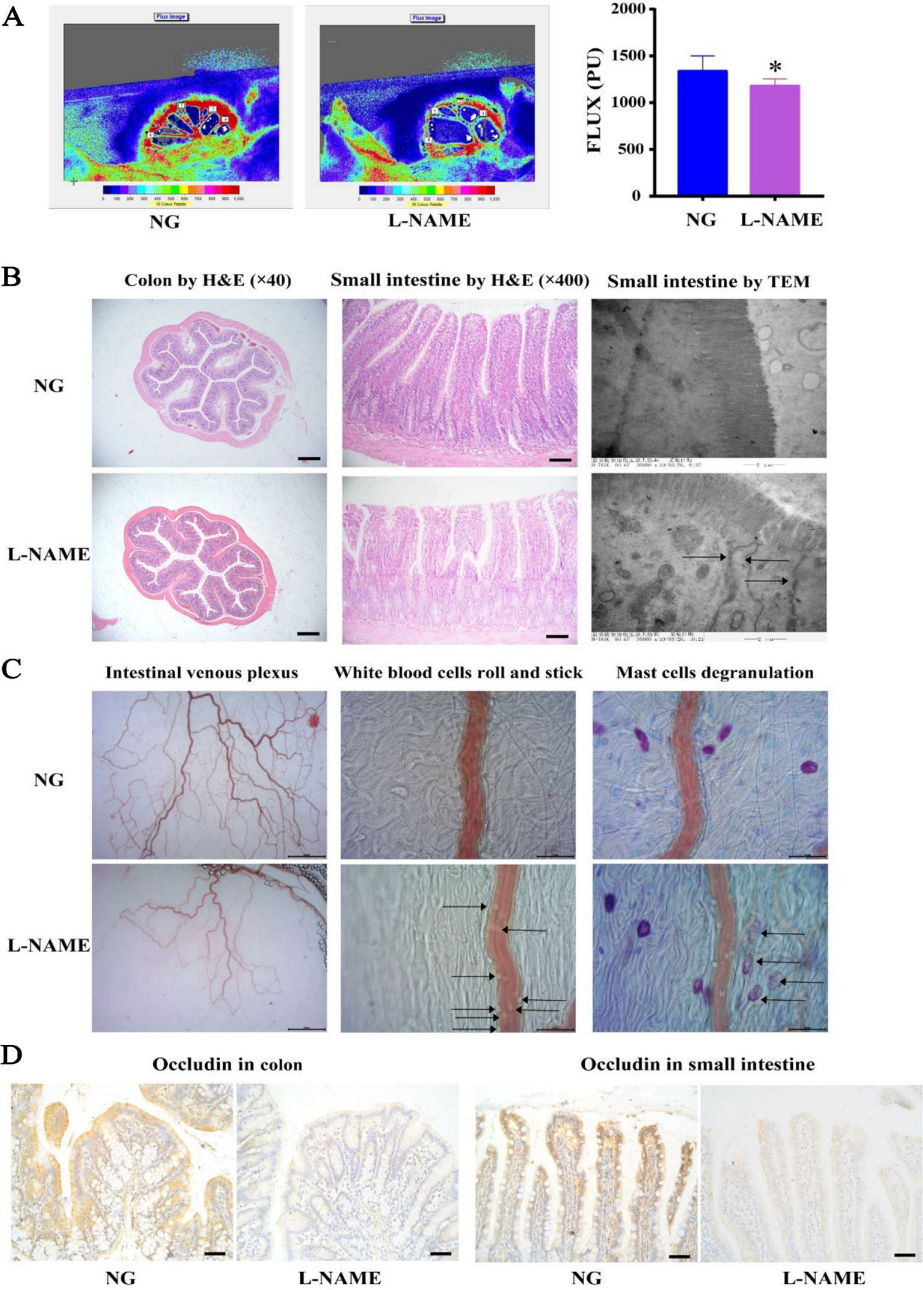
Pathophysiological alterations in the gut of the L-NAME-treated rats. **(A)** Changes of gross microcirculation and perfusion in the small intestine. **(B)** Representative photomicrograph of histological alterations in the colon (×40) and the small intestine (×200) by H&E staining, and the small intestine by transmission electron microscopy (×1,200) (the arrows indicate the disrupted tight junction proteins in the villi). **(C)** Representative photomicrograph of intestinal venous plexus, white blood cells roll and stick (the arrows), and mast cells degranulation (the arrows) in the small intestine. **(D)** Representative photomicrograph of occludin protein expression by immunohistochemistry (IHC) in the colon and the small intestine (×400). Bar = 50 μm. The data were expressed as means ± SEM. Significant differences are indicated by *P < 0.05 as compared with the NG (n = 9 per group).

There is increasing evidence of the role of host-gut interactions on hypertension and liver injury. L-NAME-induced gut microflora disorder in hypertensive rats may lead to liver lipid metabolism disorder, further aggravating liver damage. First of all, it was investigated whether the gut pathology, a significant role of host-gut interactions in the pathogenesis of hypertension, altered in the L-NAME-treated rats. It was observed that the villi in the small intestine and colon of the L-NAME-treated rats were much shorter and appeared stunted (**Figure 6B**), with the perfusion decline compared with the NG (**Figure 6A**) (*P <* 0.05). The TEM showed that villi in the L-NAME-treated rats became a shorter and looser arrangement, with a large number of disrupted tight junction proteins (TJPs) (**Figure 6B**). The TJPs, including occludin, were very important for the intestinal barrier. There were decreased on occludin expression with the IHC in the small intestine and colon of the L-NAME-treated rats (**Figure 6D**). Accordingly, using the microcirculation *in vivo* tracking and observing system, we found an increase in the white blood cells roll and stick and mast cells degranulation associations with the intestinal inflammatory status, as well as a decrease in intestinal venous plexus associations with the intestinal perfusion (**Figure 6C**). These data provided evidence to support the proposal that the L-NAME-treated rats with established hypertension have pathophysiological alterations in the gut, with a leaky intestinal barrier, an inflammation reaction, and decline of microcirculation perfusion.

**Figure 7:**
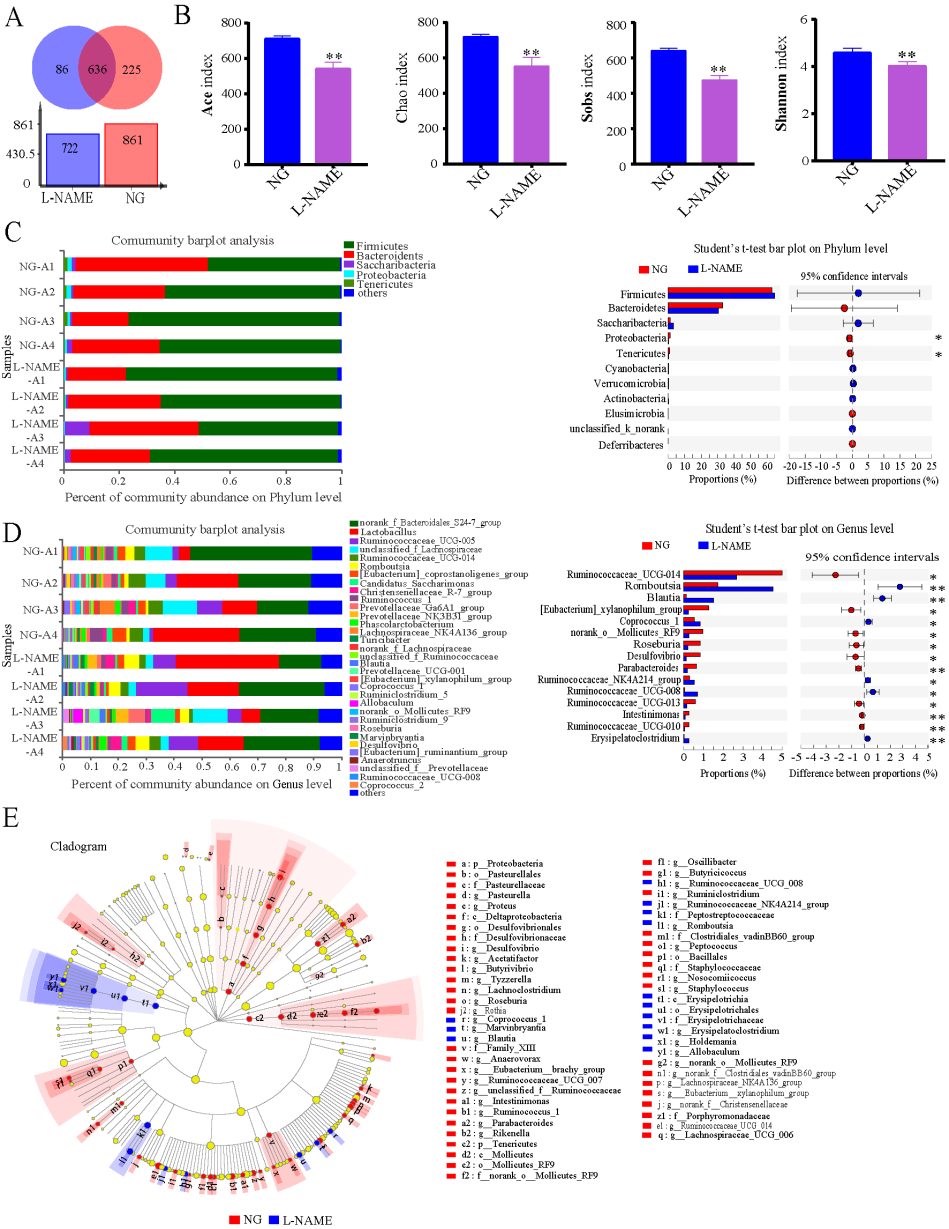
Gut microbiota dysbiosis in the L-NAME-treated rats. **(A)** The level of Operational Taxonomic Units (OTU). **(B)** Ace index, Chao index, Sobs index, and Shannon index of Alpha diversity analysis. **(C**, **D)** Percent of community abundance on Phylum and Genus levels, and relative abundances of differential bacteria analyzed by the Student’s t-test. **(E)** The taxonomic groups identified using the linear discriminant analysis effect size (LEfSe). The data were expressed as means ± SEM. Significant differences are indicated by *P < 0.05, **P < 0.01 as compared with the NG (n = 4 per group).

The above data indicate that hypertension is associated with increases in gut permeability and inflammatory status. Then to investigate whether there are also differences in the gut bacterial taxa identified through sequencing between the NG and the L-NAME-treated rats. The relative abundance of genus of all samples showed that the L-NAME treatment could decrease the gut microbiota community (**Figure 7A**), with the same decrease in the Ace index, Chao index, Sobs index, and Shannon index (**Figure 7B**) (*P < *0.01). At the phylum level, the L-NAME-treated rats associated gut microflora showed a dramatic decrease in the relative abundance of Proteobacteria and Tenericutes compared with the NG (**Figure 7C**) (*P < *0.05). Furthermore, the relative abundance of most gut microflora, such as Ruminococcaceae_UCG-014, [Eubacterium]_xylanophilum_group, norank_o_Mollicutes_RF9, Roseburia, Desulfovibrio, Parabacteroides, Ruminococcaceae_UCG-013, Intestinimonas, and Ruminococcaceae_UCG-010, showed a sharp decrease at the genus level of the L-NAME-treated rats. Also, Romboutsia, Blautia, Coprococcus_1, Ruminococcaceae_NK4A214_group, Ruminococcaceae_UCG-008, and Erysipelatoclostridium increased significantly (**Figure 7D**) (*P* < 0.05, 0.01).

The taxonomic groups that are differentially abundant between the NG and L-NAME-treated rats were identified using the linear discriminant analysis effect size (LEfSe) with α  =  0.05, LDA > 2 (**Figure 7E**). It was found out that 62 features had significantly different abundance between the NG and L-NAME-treated rats, these biomarkers accounted for 26.7% of taxonomic groups. At a genus level, gut microbiota of NG was differentially enriched with Firmicutes, Proteobacteria, Tenericutes, Actinobacteria, and Bacteroidetes, whereas the L-NAME-treated rats were differentially enriched with Firmicutes. In addition, at the generic level (LDA > 3.2), search was made for the significant differences between the NG and L-NAME-treated rats group. It was found out that Ruminococcaceae_UCG_014, Ruminococcus_1, Lachnospiraceae_NK4A136_group, Eubacterium_xylanophilum_group, unclassified_f_Ruminococceae, norank_o_Mollicutes_RF9, and Roseburia were highly enriched in the NG, while Romboutsia, Blautia, Allobaculum, and Coproccus were highly enriched in the L-NAME-treated hypertensive rats. Interestingly, it was also observed that Mollicutes was highly enriched in the NG, while Erysipelotrichia in the L-NAME-treated rats at the class level (**Figure 7E**).

### Gut Microbiota Dysbiosis Were Present Notable Correlations With Serum NO and Liver Injury Tag

The finding of our present study was that L-NAME could cause serum NO deficiency, liver microcirculation disorders, changes in liver lipid metabonomics, and microbiota dysbiosis, but it is not clear whether there are certain correlations between these changes. Therefore, we performed correlation analyses between the serum NO and intestinal bacteria, and the correlation between intestinal bacteria and liver injury tag, liver microcirculation and liver FA and TAG species, by Spearman’s correlation coefficient method.

**Figure 8:**
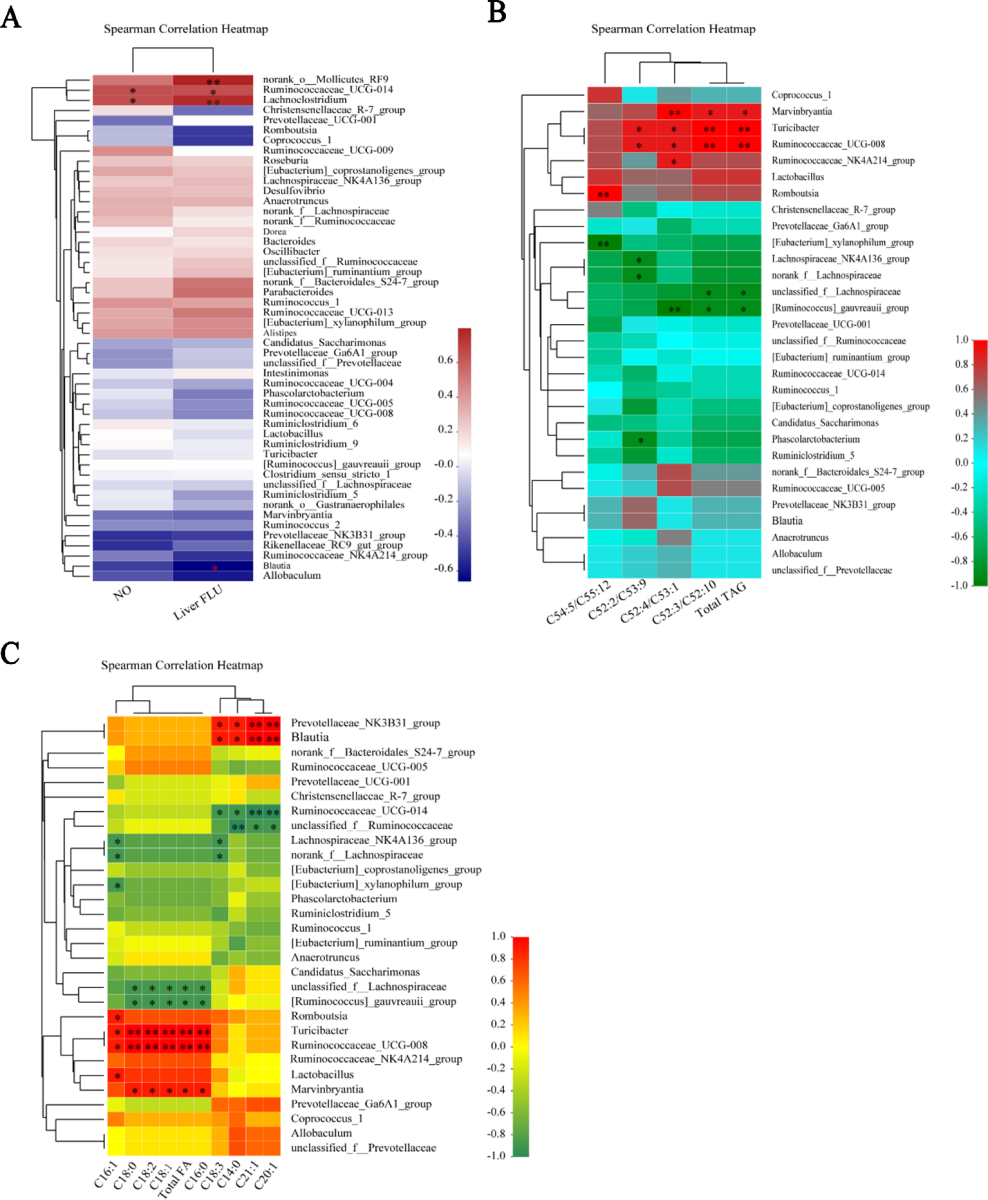
Gut microbiota dysbiosis were present notable correlations with serum NO and liver injury tag in the L-NAME-treated rats. At the genus level, the relative abundance of the gut microbial community and serum NO and liver FLUX **(A)**, TAG species **(B)**, and FA species **(C)** were presented in the Spearman’s correlation heatmap. The data were expressed as means ± SEM. Significant differences are indicated by *P < 0.05, **P < 0.01 as compared with the NG (n = 4 per group).

The correlations between the relative abundance of the gut microbial community and important metabolic parameters associated with hypertension and liver injury were analyzed Spearman’s correlation coefficient heat map. At the genus level, Ruminococcaceae_UCG-014 and Lachnoclostridium exhibited significant positive correlations with serum NO and the liver gross microcirculation perfusion (FLUX) (*P <* 0.05, 0.01). The norank_o_Mollicutes_RF9 showed significant positive correlations with the liver FLUX (*P <* 0.05). Blautia displayed the opposite trend with the liver FLUX (*P <* 0.05) (**Figure 8A**). We analyzed the relationship between the gut microbial community and liver TAG species at the genus level. Marvinbryantia, Turicibacter, Ruminococcaceae_UCG-008, Ruminococcaceae_NK4A214_group, and Romboutsia showed significant positive correlations with the liver total TAG, C52:3/C52:10, C52:4/C53:1, C52:2/C53:9, and C54:5/C55:12 (*P <* 0.05, 0.001), while [Ruminococcus]_gauvreauii_group, [Eubacterium]_xylanophilum_group, Lachnospiraceae_NK4A136_group, norank_f_Lachnospiraceae, unclassified_f_Lachnospiraceae, and Phascolarctobacterium had inverse correlations with them correspondingly (**Figure 8B**) (*P <* 0.05, 0.001).

Moreover, the relationship between the gut microbial community and liver FA species at the genus level displayed that Prevotellaceae_NK3B31_group, Blautia, Romboutsia, Turicibacter, Ruminococcaceae_UCG-008, Lactobacillus, and Marvinbryantia were significantly positive correlations with the total FA, C14:0, C16:0, C16:1, C18:0, C18:1, C18:2, C18:3, C20:1, and C21:1 (**Figure 8C**) (*P <* 0.05, 0.001), but Ruminococcaceae_UCG-014, unclassified_f_Ruminococcaceae, Lachnospiraceae_NK4A136_group, norank_f_Lachnospiraceae, [Eubacterium]_xylanophilum_group, unclassified_f_Lachnospiraceae, and [Ruminococcus]_gauvreauii_group inverse correlations with them correspondingly (**Figure 8C**) (*P <* 0.05, 0.001). It is suggested that the changes of gut microbiota may lead to the difference of liver lipid metabolism in the L-NAME-treated rats. Although there is no detailed study on how the gut microbiota affects the liver lipid metabolism, research has shown that the change of gut microbiota can affect liver fatty change, and probiotics could relieve liver lipid (Albillos et al., 2019).

## Discussion

There is sufficient available data which reveals the reciprocal relationship between hypertension and liver injury (Yehua and Xiaowa, 2000; Lyu et al., 2016; Zhu et al., 2016). It was found that L-NAME-induced NO deficiency in hypertensive rats could cause liver injury and changes in serum lipid profiles (Rajeshwari et al., 2014; Abd-Elsalam et al., 2017; Bilanda et al., 2017), as well as gut dysbiosis which could lead to liver disease (Xu et al., 2013; Albillos et al., 2019). We speculated that L-NAME-induced gut microflora disorder in hypertensive rats may lead to liver lipid metabolism disorder, further aggravating liver damage. So, this study focused on investigating the changes of liver lipometabonomics and exploring the underlying mechanisms of liver injury in the L-NAME-treated rats. It was found out that the L-NAME-induced hypertensive rats exhibiting liver injury were the joint action of hepatic abnormal fatty acid metabolism, microcirculation disorder. Further, the gut microflora, as well as the changes of enzymes in FA β-oxidation (ACOX, CPT1α), desaturation (SCD-1), and synthesis (FAS) may be the potential mechanisms for abnormal fatty acid metabolism.

The endothelium-derived vasodilatory factor, NO, is a widespread biological mediator involved in many physiological and pathological processes and a crucial regulator of vascular tone (Zhu et al., 2016; Li et al., 2018a). L-NAME, an established NO synthase inhibitor, is known to induce hypertension characterized by endothelial dysfunction along with marked deficiency of NO (Seth et al., 2016). In our study, the level of biochemical parameter NO was significantly decreased in the serum and BP was significantly increased of the L-NAME-treated group, similar to other research findings. In the present study, SBP, DBP, and MBP increased in the L-NAME-treated rats, the eNOS-NO pathway was disordered with the expression of eNOS in aorta endothelium, and serum NO decreased. These results showed that the BP of the model rats increased significantly, which appears to simulate the state of vascular hypertension in patients.

For further investigation of the liver injury caused by L-NAME, the serum ALT and AST levels were recorded. When a tissue is damaged, AST and ALT enzymes leak out of the cells into the blood primarily from mitochondria or the cytoplasm. Thus, they serve as important markers for tissue damage. ALT is produced mainly in the liver, while AST is produced in heart, kidney, liver, and muscle tissues. An increase in the level of these enzymes helps predict the type and extent of tissue damage. In particular, liver tissue damage is closely associated with the elevated serum ALT level (Seth et al., 2016). In the present study, liver injury with L-NAME-induced hypertension measured by a significant increase in the levels of serum ALT is demonstrated. Hypertension is not only an abnormal hemodynamic disease, but also a metabolic disease which may coexist with lipid, glucose, and other metabolic disorders (Jian, 2002). As follows from the L-NAME-induced hypertensive rats exhibited elevated serum liver enzymes accompanied by elevated blood lipids (Seth et al., 2016; Silambarasan et al., 2016). In our study, it was also found that the levels of serum TC and TG in the L-NAME-treated rats were significantly increased.

Additionally, changes in hepatic microcirculation and histopathology were also studied to assess the extent of liver injury. The liver is a blood-rich organ, and NO is very important to maintain the function of liver microvessels. The regulated eNOS-NO pathway protects ischemia-reperfusion liver injury *via* activating eNOS, thus increasing NO, which protects hepatocytes from the insults (Duan et al., 2017). L-arginine, a prerequisite of NO, is a useful adjunct for preventing hepatic injury after trauma-hemorrhage *via* endothelial-derived NO production (Angele et al., 2000). In this study, it was observed that the gross hepatic microcirculation of the L-NAME-treated rats was significantly reduced. The further examination of microcirculation *in vivo* showed that the blood flow and vascular richness of the liver margin were significantly decreased. This finding suggested that L-NAME can create NO deficiency and can cause reduced hepatic blood flow, which can damage the liver and liver function.

The liver injury is described by a slight mononuclear infiltration (Seth et al., 2016), increase glycogen content (Tarsitano et al., 2007), vascular remodeling (Chen et al., 2019), and abnormal lipid accumulation (Silambarasan et al., 2016) in the long-term L- NAME-treated rats. In the present study, histological alterations in the hepatic tissue were studied to assess the extent of liver damage using the H&E and OR-stained liver sections. There was a wide range of abnormal lipid accumulation, including many small lipid droplets, in the liver of the L-NAME-treated rats. TEM was used to examine the ultrastructure of hepatocytes, which showed that the hepatocytes exhibited lipid droplets accumulation, mitochondrial damage, and a damaged endoplasmic reticulum structure.

The change in the proportion of saturated/unsaturated FA is a potential biomarker for chronic metabolic diseases (Huang et al., 2019). In recent years, the relationship between the hepatic fatty acid metabolism and the liver injury has been investigated (Huang et al., 2019). Our study found that L-NAME could significantly increase most of the TAG and FA in the liver analyzed with MDMS-SL. The further study of the effect of L-NAME on fatty acid metabolism-related proteins in the liver, fatty acid synthesis (FAS), uptake (FATP2), desaturase (SCD-1), and oxidative decomposition related proteins (ACOX1 and CPT1α) in the liver were measured. FATP2 is a member of the FATP family of fatty acid uptake mediators. Earlier research has found that FATP2 (Slc27a2) is expressed in specific tissue types, particularly the small intestine, pancreas, and liver (Falcon et al., 2010; Sanchez et al., 2017). These studies showed that the expression of FATP2 in the liver is consistent with its primary role in the fatty acid transport and metabolism. There is a positive correlation between FATP2 and the progression of liver injury (Krammer et al., 2011). SCD-1, a key rate-limiting enzyme in the desaturation of fatty acid, was significantly elevated in the liver of obese mice on a high-fat diet (Huang et al., 2019), and it is a potential therapeutic target for the liver injury (Shinohara et al., 2012; Huang et al., 2019). FAS, a key enzyme in fatty acid synthesis, catalyzes acetyl coenzyme A and malonyl coenzyme A to produce long-chain fatty acids. The expression of FAS was increased in the injured HepG2 cells and the KK-A^y^ mice livers with lipid metabolic disorders (Li et al., 2018b). Other research shows that L-NAME treatment significantly increased the activity of 3-hydroxy-3-methylglutaryl-Coenzyme A (HMG-CoA) reductase in the liver to induce lipid accumulation (Silambarasan et al., 2016). The results in our study showed that L-NAME increased SCD-1 and FAS expression in the liver, but not FATP2, which is consistent with the increase of total hepatic lipids. These findings suggested that the changes in the hepatic fatty acid metabolism by L-NAME are related to the increase in fatty acid desaturation and synthesis.

Mitochondria of hepatocytes decompose free FA by β-oxidation. In this process, the rate-limiting enzymes are mainly CPTlα and ACOX1 (Huang et al., 2019). ACOX1 is an enzyme related to FA oxidation in adipocytes, and also is the initiating enzyme of the β-oxidation system in peroxidase (Nakade et al., 2017). The studies have shown that a methionine-choline-deficient diet can attenuate ACOX1 levels in the liver of NASH mice and that ACOX1 deficient mice could develop severe liver injury (Dong et al., 2019). Carnitine palmitoyl transferase 1 (CPT1) is one of the carnitine palmitoyl transferases (CPT) and a rate-limiting enzyme in the oxidation of fatty acid-beta (Guo et al., 2016). A decrease of CPT1 expression could lead to a decrease of acyl coenzyme A transport to mitochondria. This decrease induces liver injury resulting in the deposition of fatty acid and acyl coenzyme A in the cytoplasm (Wang, 2018). Our results showed that L-NAME treatment could downregulate the expression of CPTlα and ACOX1 in the liver. These findings suggested that the changes in hepatic fatty acid metabolism by L-NAME is related to the suppression in fatty acid β-oxidation and the increase in fatty acid desaturation and synthesis, results in increase of the liver total fatty acid content in the L-NAME-treated rats.

It has become evident in recent years that the host-gut interaction is related to hypertension (Santisteban et al., 2017). Some research has demonstrated that there are gut pathophysiological alterations in the hypertensive rats, mice, and humans (Yang et al., 2015; Li et al., 2017b; Santisteban et al., 2017). The gut pathophysiological alterations have been related to many other diseases, including dyslipidemia and liver injury (Cremonini et al., 2019). In this study, we investigated whether the L-NAME treatment alters intestinal pathophysiology in rats with the changes of hepatic fatty acid metabolism. These data showed that the L-NAME-treated rats have pathophysiological alterations in the gut, such as a leaky intestinal barrier and an inflammation reaction, which is also present in the SHR and chronic angiotensin II infusion rats (Santisteban et al., 2017). An interesting observation is a significant decrease in mesenteric blood flow of the L-NAME-treated rats which is consistent with the reports indicating that the decreased gut blood flow is associated with the gut pathophysiology alterations and hypertension (Granger et al., 2015; Santisteban et al., 2017).

It is now widely accepted that gut microbiota is an important environmental factor involved in the pathogenesis of hypertension and liver disease (Yang et al., 2015; Li et al., 2017b; Santisteban et al., 2017; Albillos et al., 2019). Our study found that after the treatment with L-NAME diversity and abundance of the gut microflora decreased and the number of some harmful bacteria in the L-NAME-treated rats significantly increased. Several studies have found a correlation between Blautia and obesity. For example, Goffredo et al. reported a positive correlation between the abundance of Blautia and obesity in American youth and verified that the level of acetate, which is the product by Blautia, is associated with the body fat partitioning and hepatic lipogenesis (Goffredo et al., 2016). It was found out that Ruminococcaceae_UCG-014, Lachnoclostridium, and Blautia exhibited significant correlations with the liver FLUX as well as Ruminococcaceae_UCG-014 and Lachnoclostridium significant correlations the decrease of serum NO level in the L-NAME-induced hypertensive rats in our study. Meanwhile, the L-NAME-induced hypertensive rats revealed pathophysiological alterations in the gut with a leaky intestinal barrier, inflammation reaction, and decline of microcirculation perfusion, as well as gut microbiota dysbiosis. Moreover, there was a significant correlation between the gut microbiota dysbiosis and the decrease of serum NO level as well as liver injury tag, liver microcirculation and liver FA and TAG species in the L-NAME-induced hypertensive rats. Other research has shown that exogenous NO supplementation can improve LPS induced intestinal damage and intestinal bacteria disorder (Cuzzolin et al., 1997), which shows that NO plays an important role in the intestinal pathophysiology. It was found out that the decrease of endogenous NO could lead to these changes of intestinal pathophysiology, especially intestinal bacteria disorder, could be a significant factor in the initiation and establishment of hypertension and associated liver injury as well as lipid metabolic disorder.

In conclusion, this study found that the L-NAME-induced hypertensive rats exhibited liver injury which was the joint action of hepatic abnormal fatty acid metabolism, and microcirculation disorder. Further, the gut microflora, as well as the changes of fatty acid β-oxidation (ACOX, CPT1α), desaturation (SCD-1), and synthesis (FAS) may be the potential mechanisms for abnormal fatty acid metabolism. The limit of our research was that we clarified, using innovative methods, that gut microflora was significantly correlated to liver lipidomics in hypertension rats, but did not show the mechanism.

## Data Availability Statement

The datasets analyzed in this manuscript are not publicly available. Requests to access the datasets should be directed to boli19861023@163.com.

## Ethics Statement

The animal study was reviewed and approved by the Ethics Committee of Zhejiang University of Technology.

## Author Contributions

G-YL and S-HC conceptualized the study and designed the research. S-SL, BL, N-YZ, Y-HC, F-CZ, and XH conducted the experiments. BL, S-SL, JS, J-J Y, L-ZL, XZ, Y-ZW, XH, RL, SX, and DK performed the acquisition and analysis of data. S-SL, BL, and DK wrote the manuscript with the input of other co-authors.

## Funding

This study was supported by the National Science Foundation of China (No.81803760, No. 81873036, No. 81673638, No. 81874352, No. 81703772, and No. 81803819), the National Science Foundation of Zhejiang Province (No. LQ18H280003, No. LQ17H280004, and LQ17H280005), the National Key Research and Development Program (No. 2017YFC1702200), the China Postdoctoral Science Foundation (No. 2018M632506), and the Key Research and Development Program of Zhejiang Province (No. 2017C03052 and No. 2015C02032).

## Conflict of Interest

The authors declare that the research was conducted in the absence of any commercial or financial relationships that could be construed as a potential conflict of interest.
